# Association Between First-Line Immune Checkpoint Inhibition and Survival for Medicare-Insured Patients With Advanced Non–Small Cell Lung Cancer

**DOI:** 10.1001/jamanetworkopen.2021.11113

**Published:** 2021-05-21

**Authors:** Kenneth L. Kehl, Scott Greenwald, Nassib G. Chamoun, Paul J. Manberg, Deborah Schrag

**Affiliations:** 1 Division of Population Sciences, Department of Medical Oncology, Dana-Farber Cancer Institute, Harvard Medical School, Boston, Massachusetts; 2 Health Data Analytics Institute, Dedham, Massachusetts; 3 Associate Editor, *JAMA*

## Abstract

**Importance:**

Immunotherapy is now a cornerstone of treatment for advanced non–small cell lung cancer (NSCLC), but its uptake and effectiveness among older patients outside clinical trials remain poorly understood.

**Objective:**

To understand treatment patterns and evaluate the overall survival associated with checkpoint inhibitor immunotherapy, cytotoxic chemotherapy, and combined chemoimmunotherapy for older patients who have advanced NSCLC and Medicare coverage.

**Design, Setting, and Participants:**

This retrospective cohort study included Medicare-insured patients in the US aged 66 to 89 years who initiated first palliative-intent systemic therapy for lung cancer between January 1, 2016, and December 31, 2018. Survival follow-up continued through March 31, 2020. A total of 19 529 patients who had advanced lung cancer and were insured by a Medicare fee-for-service plan were included in the analysis.

**Exposures:**

Regimens included pembrolizumab monotherapy (n = 3079), combined platinum-based drug (ie, cisplatin or carboplatin [hereinafter, platinum]) and pemetrexed disodium (n = 5159), combined platinum and a taxane (ie, paclitaxel, nab-paclitaxel, or docetaxel) (n = 9866), and combined platinum, pemetrexed, and pembrolizumab (n = 1425), as ascertained using Medicare claims from the Centers for Medicare & Medicaid Services.

**Main Outcomes and Measures:**

The primary outcome was overall survival, which was measured using the restricted mean survival time (RMST) with propensity score adjustment for clinical and sociodemographic characteristics. Median survival was also reported for comparison with outcomes from registrational trials.

**Results:**

A total of 19 529 patients (54% male, 46% female; median age, 73.8 [interquartile range, 69.9-78.4] years) were identified for analysis. The uptake of pembrolizumab-containing regimens in the Medicare population was rapid, increasing from 0.7% of first-line treatments in the second quarter of 2016 to 42.4% in the third quarter of 2018. Patients who were older (≥70 years, 2484 [81%]), were female (1577 [51%]), and/or had higher Risk Stratification Index scores (highest quintile, 922 [30%]) were more likely to receive single-agent pembrolizumab than chemotherapy. After propensity score adjustment, pembrolizumab was associated with survival similar to platinum/pemetrexed (RMST difference, −0.2 [95% CI, −0.5 to 0.2] months) or platinum/taxane (RMST difference, −0.7 [95% CI, −1.0 to −0.4] months). Patients receiving platinum/pemetrexed/pembrolizumab chemoimmunotherapy also had adjusted survival similar to those receiving platinum/pemetrexed chemotherapy (RMST difference, 0.5 [95% CI, 0.1-0.9] months). The unadjusted median survival was 11.4 (95% CI, 10.5-12.3) months among patients receiving single-agent pembrolizumab, approximately 15 months shorter than observed among pembrolizumab-treated participants in the KEYNOTE-024 trial. The unadjusted median survival was 12.9 (95% CI, 11.8-14.0) months among patients receiving platinum/pemetrexed/pembrolizumab chemoimmunotherapy, approximately 10 months shorter than observed among platinum/pemetrexed/pembrolizumab–treated participants in the KEYNOTE-189 trial.

**Conclusions and Relevance:**

Immunotherapy has been incorporated rapidly into treatment for patients with advanced NSCLC. However, survival estimates in the Medicare population are much shorter than those reported in registrational trials. These results provide contemporary estimates of survival for older patients with advanced NSCLC treated in routine practice, facilitating patient-centered decision-making.

## Key Points

**Question:**

Has the incorporation of checkpoint inhibitor immunotherapy into initial treatment of older Medicare-insured patients with advanced non–small cell lung cancer been associated with meaningful improvements in overall survival?

**Findings:**

This cohort study included 19 529 patients s with Medicare coverage who initiated first systemic therapy for advanced lung cancer using 1 of 4 regimens of checkpoint inhibitor immunotherapy, cytotoxic chemotherapy, and combined chemoimmunotherapy. The median overall survival was 11.4 months among patients receiving pembrolizumab monotherapy and 12.9 months among patients receiving platinum/pemetrexed/pembrolizumab chemoimmunotherapy, both substantially shorter than observed in registrational trials, with an adjusted restricted mean survival time through 18 months of follow-up of 11 to 12 months for all 4 treatment groups.

**Meaning:**

In this study, immunotherapy among older Medicare-insured patients with advanced non–small cell lung cancer was associated with shorter overall survival than observed in key clinical trials, providing patients and physicians with estimates of outcomes for older patients who have lung cancer and are being treated with immunotherapy.

## Teaser

This cohort study assesses the treatment patterns of and survival outcomes associated with checkpoint inhibitor immunotherapy, cytotoxic chemotherapy, and combined chemoimmunotherapy for older patients with advanced non–small cell lung cancer and Medicare coverage.

## Introduction

Immunotherapy has rapidly changed the therapeutic landscape for patients with cancer. Since the approval of ipilimumab in 2011 for the treatment of metastatic melanoma,^1^ pembrolizumab, nivolumab, atezolizumab, avelumab, and durvalumab have been approved for advanced melanoma,^2,3,4,5,6,7,8^ renal cell carcinoma,^9^ Hodgkin lymphoma,^10^ Merkel cell carcinoma,^11^ urothelial carcinoma,^12^ head and neck cancer,^13^ cervical cancer,^14^ gastric cancer,^15^ breast cancer,^16^ and lung cancer.^17,18,19,20,21,22^ Pembrolizumab has also been approved for tumors with high microsatellite instability, independent of histologic findings.^23^ There are now hundreds of thousands of patients diagnosed annually in the US with a cancer for which checkpoint inhibitors are indicated.^24^

The population-level implications of this shift are most evident for lung cancer, which remains the leading cause of cancer death.^24^ Pembrolizumab was the first checkpoint inhibitor approved in the US for the first-line treatment of advanced non–small cell lung cancer (NSCLC), based initially on 2 key trials. The KEYNOTE-024 trial randomized patients with tumors in which programmed cell death 1 ligand-1 (PD-L1) expression levels were at least 50% to pembrolizumab or platinum-based (hereinafter, platinum) chemotherapy, finding that first-line pembrolizumab substantially improved overall survival in this population (median, 30.0 vs 14.2 months with chemotherapy).^25,26^ The KEYNOTE-189 trial randomized patients with metastatic nonsquamous NSCLC, regardless of tumor PD-L1 status, to first-line combined platinum, pemetrexed disodium, and pembrolizumab chemoimmunotherapy vs combined platinum and pemetrexed chemotherapy alone; the addition of pembrolizumab improved overall survival (median, 22.0 vs 10.7 months with chemotherapy).^27,28^

Based on these results, practice guidelines now recommend up-front immunotherapy for patients with advanced NSCLC; however, treatment patterns and outcomes among older patients who may not have met inclusion criteria for key clinical trials remain unclear. Historically, older adults and patients with comorbid conditions and frailty have been underrepresented in cancer clinical trials,^29,30^ such that it is unclear to what extent clinical trial results generalize to these populations. Furthermore, immunotherapy benefit patterns among relevant subgroups may vary; for example, a meta-analysis of randomized clinical trials across primary tumor sites^31^ suggested that immune checkpoint inhibitors may confer less benefit to women than to men.

Because most of the approximately 230 000 patients diagnosed with lung cancer each year in the US are eligible for Medicare,^32^ understanding practice pattern trends and outcomes associated with introduction of pembrolizumab therapy in the Medicare-insured population is important. Such data may inform prognostic discussions in clinical practice; moreover, evaluation of Medicare claims may expeditiously identify subgroups who obtain unusually large or small survival benefits from treatment. The primary objective of this analysis was to understand treatment patterns and compare the survival outcomes associated with immunotherapy (pembrolizumab), cytotoxic chemotherapy (platinum/pemetrexed or platinum with a taxane [ie, paclitaxel, nab-paclitaxel, or docetaxel]), and combination chemotherapy and immunotherapy (platinum/pemetrexed/pembrolizumab) when used for initial treatment of newly diagnosed advanced NSCLC with palliative intent in older patients with Medicare fee-for-service coverage.

## Methods

### Data Source

Data were obtained from the Centers for Medicare & Medicaid Services (CMS) under a data use agreement. *International Classification of Diseases, Ninth Revision, Clinical Modification*, codes for observations before October 2015 were mapped into their corresponding *International Statistical Classification of Diseases, Tenth Revision, Clinical Modification* (*ICD-10-CM*), codes using general equivalence mappings available from CMS.^33^ This study was determined to be exempt from informed consent by the New England Independent Review Board, which approved the study, and all unique identifiers of participants were encrypted. The study was conducted in accordance with the Strengthening the Reporting of Observational Studies in Epidemiology (STROBE) reporting guideline.^34^

### Study Cohort

The analysis was conducted using Medicare fee-for-service claims from January 1, 2015, through March 31, 2020. The key inclusion criteria were an index claim for an *ICD-10-CM* lung cancer diagnosis code from 2016 to 2018 and administration of one of the following combinations of systemic therapy, in which the components of chemotherapy began on the same day, and immunotherapy within 3 weeks of chemotherapy if included in a composite combination, all within 3 months of that index claim: (1) pembrolizumab (Healthcare Common Procedural Coding System [HCPCS] code C9027 or J9271); (2) platinum (HCPCS code J9060 or J9045) and pemetrexed (HCPCS code J9305); (3) platinum and taxane (HCPCS code J9267, J9264, or J9171); or (4) platinum, pemetrexed, and pembrolizumab.

Additional inclusion criteria comprised age 66 to 89 years at the time of the index claim; treatment onset at least 18 months before the end of follow-up on March 31, 2020; and continuous Medicare parts A and B fee-for-service coverage for at least 12 months before the index lung cancer claim (to determine comorbidity risk scores and derive a first-line treatment cohort) and until death or 3 months after the index claim, whichever came first. Exclusion criteria consisted of (1) any Medicare part C coverage in the 12 months before or 3 months after the index claim, which would have rendered assessment of delivered health care services unreliable; (2) any prior claim for a lung cancer diagnosis in the 12 months before the index claim; (3) any claim for a diagnosis of another primary cancer, defined using Clinical Classification Software,^35^ in the 12 months before or 3 months after the index claim; and (4) any lung surgery (other than biopsy) or concurrent chemotherapy and radiotherapy from 3 months before to 3 months after the index claim. These exclusions were applied because those treatments are recommended for early-stage lung cancer,^36^ and the goal in this analysis was evaluation of systemic treatment for advanced disease. An exploratory sensitivity analysis was also performed among patients with part D prescription drug coverage, in which patients who received oral targeted therapies (gefitinib, erlotinib hydrochloride, afatinib dimaleate, dacomitinib, osimertinib mesylate, crizotinib, ceritinib, brigatinib, alectinib hydrochloride, lorlatinib, dabrafenib mesylate, or trametinib dimethyl sulfoxide) at any time from 12 months before the index claim through the end of follow-up were excluded. Exploratory subgroup analyses by sex and age were also performed.

### Survival Outcome

The primary outcome was overall survival from treatment initiation, which was ascertained using the restricted mean survival time (RMST) through 18 months of follow-up.^37^ Differences in RMST were selected as the outcome metric rather than the commonly used hazard ratio, because in checkpoint inhibitor studies, the proportional hazards assumption may be violated owing to a pattern of durable benefit among a subgroup of patients receiving immunotherapy.^38^ Unadjusted median survivals were also calculated to facilitate comparison of outcomes with those observed in clinical trials. Survival was obtained from the Medicare beneficiary file, with a cutoff date of March 31, 2020.

### Treatment Exposures and Covariates

The key independent variable for analysis was the first-line systemic therapy regimen. Regimens included (1) pembrolizumab immunotherapy, (2) platinum/pemetrexed chemotherapy, (3) platinum/taxane chemotherapy, and (4) platinum/pemetrexed/pembrolizumab chemoimmunotherapy. These regimens were chosen because they were recommended for NSCLC but not SCLC, per National Comprehensive Cancer Network guidelines.^39,40^ Covariates included age; sex; race/ethnicity as defined by Medicare; any dual Medicaid enrollment in the 12 months before lung cancer diagnosis; the median household income in each patient’s census tract of residence, categorized in quintiles; the urban/rural status of each patient’s county of residence, defined using the 2013 Rural Urban Continuum Code for each patient’s county^41^; the date of initial systemic lung cancer therapy, categorized into quarters (ranging from the first quarter of 2016 to the last quarter of 2018); the proportion of adults with a college degree in each patient’s zip code of residence, categorized in quintiles; and estimates of 1-year mortality, derived using the Risk Stratification Index^42,43^ (RSI) for the date of initial treatment using the *ICD-10-CM* codes recorded in the year before the estimated date, categorized in quintiles.

### Statistical Analysis

A multivariable RMST regression model^37^ was created to explore associations between each treatment strategy and overall survival. Next, to conduct binary comparisons between individual treatment strategies, propensity score adjustment models were created. The aforementioned covariates were included in models a priori. Three models were created to calculate the propensity to receive (1) pembrolizumab (vs platinum/pemetrexed), (2) pembrolizumab (vs platinum/taxane), and (3) platinum/pemetrexed/pembrolizumab (vs platinum/pemetrexed). Next, treatment comparisons were performed using RMST regression, with propensity score stratification into quintiles.^44^ Two-sided *P* < .05 was considered statistically significant; exploratory subgroup and sensitivity analyses were not adjusted for multiple comparisons. Hypothesis tests were 2 sided. Analyses were performed in SAS Enterprise Guide, version 7.15 (SAS Institute).

## Results

**Figure 1.**
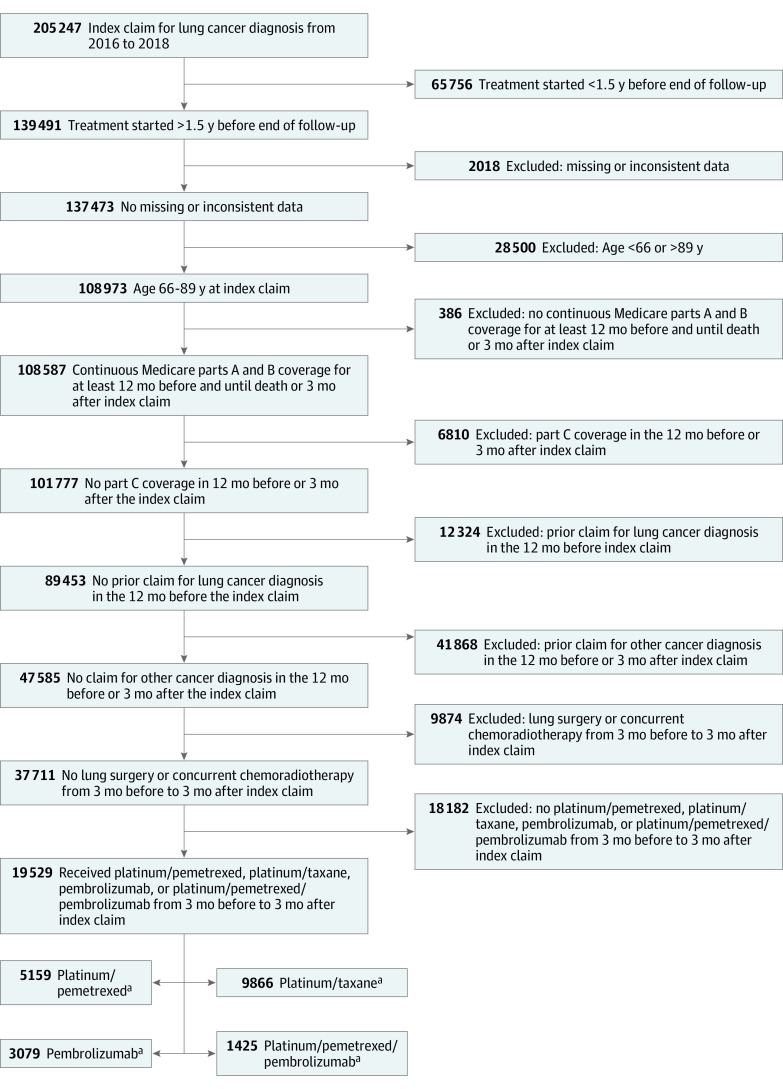
Cohort Derivation ^a^A treatment regimen was defined as any combination of chemotherapy drugs, the first dose of each of which was administered within 21 days of the first dose of the first drug given in the regimen. Platinum was defined as cisplatin or carboplatin; taxane, as paclitaxel, nab-paclitaxel, or docetaxel. Coadministration of bevacizumab was not considered to define a distinct regimen.

Between January 1, 2016, and December 31, 2018, 19 529 patients met study selection criteria (Figure 1 and eFigure 1 in the Supplement); 54% of patients were male, 46% were female, and the median age was 73.8 (interquartile range, 69.9-78.4) years. Of the 19 529 patients, 3079 received pembrolizumab immunotherapy; 5159 received chemotherapy with platinum/pemetrexed; 9866 received chemotherapy with platinum/taxane; and 1425 received chemoimmunotherapy with platinum/pemetrexed/pembrolizumab. The uptake of pembrolizumab-containing regimens was rapid, accounting for 0.7% of these first-line treatments in the second quarter of 2016 and increasing to 42.4% in the third quarter of 2018. Compared with patients treated with chemotherapy, patients treated with pembrolizumab alone were more likely to be female (1577 [51%]) and older (≥70 years, 2484 [81%]) and to have higher RSI scores (signifying a higher burden of comorbid illness; 922 [30%]) at the time of treatment (Table 1 and eTables 1 and 2 in the Supplement).

**Table 1.** Baseline Cohort Characteristics by Treatment Group

Characteristic	No. (%) of patients				
	All (N = 19 529)	First-line treatment group			
		Platinum/pemetrexed (n = 5159)	Platinum/taxane (n = 9866)	Pembrolizumab (n = 3079)	Platinum/pemetrexed/pembrolizumab (n = 1425)
Age at the index lung cancer event, y					
66-69	4963 (25)	1538 (30)	2430 (25)	595 (19)	400 (28)
70-79	11 012 (56)	2921 (57)	5680 (58)	1571 (51)	840 (59)
80-89	3554 (18)	700 (14)	1756 (18)	913 (30)	185 (13)
Sex					
Male	10 509 (54)	2623 (51)	5641 (57)	1502 (49)	743 (52)
Female	9020 (46)	2536 (49)	4225 (43)	1577 (51)	682 (48)
Race/ethnicity					
Non-Hispanic					
White	17 305 (89)	4555 (88)	8734 (89)	2738 (89)	1278 (90)
Black	1395 (7)	352 (7)	773 (8)	184 (6)	86 (6)
Hispanic	106 (1)	35 (1)	46 (0.5)	19 (1)	NA^a^
Other	723 (4)	217 (4)	313 (3)	138 (4)	NA^a^
Medicaid enrollment					
No	14 993 (77)	4174 (81)	7243 (73)	2413 (78)	1163 (82)
Yes	4536 (23)	985 (19)	2623 (27)	666 (22)	262 (18)
Median household income in the census tract of residence, quintile					
Lowest 20%	3905 (20)	892 (17)	2219 (22)	551 (18)	243 (17)
20%-40%	3906 (20)	924 (18)	2154 (22)	559 (18)	269 (19)
40%-60%	3906 (20)	998 (19)	2017 (20)	604 (20)	287 (20)
60%-80%	3905 (20)	1068 (21)	1883 (19)	650 (21)	304 (21)
Highest 20%	3907 (20)	1277 (25)	1593 (16)	715 (23)	322 (23)
Urban/rural status of patient’s county					
Missing	0	0	0	0	0
Large metropolitan	8031 (41)	2410 (47)	3604 (37)	1363 (44)	654 (46)
Metropolitan	6822 (35)	1722 (33)	3579 (36)	1073 (35)	448 (31)
Urban	1668 (9)	379 (7)	953 (10)	227 (7)	109 (8)
Less urban	2417 (12)	524 (10)	1397 (14)	331 (11)	165 (12)
Rural	591 (3)	124 (2)	333 (3)	85 (3)	49 (3)
Date of initial systematic lung cancer treatment					
2016 first quarter	1071 (5)	422 (8)	641 (6)	NA^a^	NA^a^
2016 second quarter	1780 (9)	668 (13)	1108 (11)	NA^a^	NA^a^
2016 third quarter	1792 (9)	692 (13)	1094 (11)	NA^a^	NA^a^
2016 fourth quarter	1674 (9)	582 (11)	918 (9)	173 (6)	NA^a^
2017 first quarter	1883 (10)	569 (11)	930 (9)	384 (12)	NA^a^
2017 second quarter	1902 (10)	432 (8)	929 (9)	435 (14)	106 (7)
2017 third quarter	1865 (10)	389 (8)	903 (9)	388 (13)	185 (13)
2017 fourth quarter	1782 (9)	402 (8)	806 (8)	382 (12)	192 (13)
2018 first quarter	1909 (10)	400 (8)	819 (8)	464 (15)	226 (16)
2018 second quarter	1916 (10)	330 (6)	872 (9)	399 (13)	315 (22)
2018 third quarter	1883 (10)	266 (5)	818 (8)	416 (14)	383 (27)
2018 fourth quarter^b^	72 (0.4)	NA	NA	NA	NA
Adults with college degree in patient’s zip code of residence, quintile					
Lowest 20%	3931 (20)	851 (16)	2312 (23)	513 (17)	255 (18)
20%-40%	3914 (20)	958 (19)	2116 (21)	567 (18)	273 (19)
40%-60%	3880 (20)	1020 (20)	1988 (20)	605 (20)	267 (19)
60%-80%	3912 (20)	1126 (22)	1855 (19)	636 (21)	295 (21)
Highest 20%	3892 (20)	1204 (23)	1595 (16)	758 (25)	335 (24)
RSI at baseline, quintile					
Lowest 20%	3905 (20)	1061 (21)	2227 (23)	384 (12)	233 (16)
20%-40%	3907 (20)	970 (19)	2200 (22)	473 (15)	264 (19)
40%-60%	3905 (20)	1052 (20)	1958 (20)	598 (19)	297 (21)
60%-80%	3907 (20)	1046 (20)	1848 (19)	702 (23)	311 (22)
Highest 20%	3905 (20)	1030 (20)	1633 (17)	922 (30)	320 (22)

Abbreviations: NA, not applicable; RSI, Risk Stratification Index.

^a^ Cell information suppressed consistent with Centers for Medicare & Medicaid Services cell size suppression policy.

^b^ Data for 2018 fourth quarter are incomplete.

**Figure 2.**
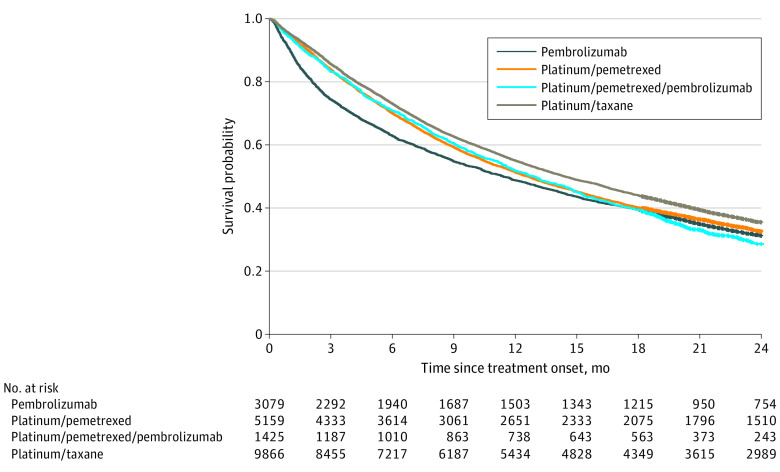
Unadjusted Kaplan-Meier Survival Curves by Treatment Group for All Participants The pembrolizumab-treated group had 2038 events and 771 censored patients (median survival, 11.4 [95% CI, 10.5-12.3] months); the platinum/pemetrexed–treated group, 4032 events and 1127 censored patients (median survival, 12.6 [95% CI, 12.0-13.1] months); the platinum/permetrexed/pembrolizumab–treated group, 1054 events and 371 censored patients (median survival, 12.9 [95% CI, 11.8-14.0] months); and the platinum/taxane–treated group, 7349 events and 2517 censored patients (median survival, 14.4 [95% CI, 13.9-14.9] months).

The unadjusted median survival among patients receiving single-agent pembrolizumab was 11.4 (95% CI, 10.5-12.3) months, which was approximately 15 months shorter than reported in the KEYNOTE-024 trial.^25,26^ The unadjusted median survival among patients receiving platinum/pemetrexed/pembrolizumab chemoimmunotherapy was 12.9 (95% CI, 11.8-14.0) months, which was approximately 10 months shorter than reported in KEYNOTE-189.^28^ In unadjusted analyses, patients who received single-agent pembrolizumab had inferior overall survival early after initiating treatment compared with patients who received chemotherapy, but this difference diminished over time (ie, the gap between Kaplan-Meier curves narrowed with longer follow-up [Figure 2]). Unadjusted and adjusted associations between all independent variables of interest and overall survival are detailed in Table 2. The RSI scores were strongly associated with outcomes, as expected (unadjusted RMST among patients in the highest RSI quintile, 8.1 [95% CI, 7.9-8.3] months vs 14.2 [95% CI, 14.0-14.4] months among patients in the lowest quintile; *P* < .001) (eFigure 2 in the Supplement).

**Table 2.** Associations Between Patients’ Sociodemographic Characteristics and Overall Survival

Characteristic	Unadjusted models, RMST (95% CI)	Adjusted models		
		Adjusted RMST (95% CI)	Adjusted RMST difference (95% CI)	*P* value
First-line systemic treatment				
Platinum/pemetrexed	11.2 (11.1 to 11.4)	11.2 (10.7 to 11.7)	1 [Reference]	NA
Platinum/taxane	11.8 (11.6 to 11.9)	11.6 (11.0 to 12.1)	0.4 (0.2 to 0.6)	<.001
Pembrolizumab alone	10.4 (10.2 to 10.7)	10.8 (10.2 to 11.4)	−0.4 (−0.7 to −0.1)	.007
Platinum/pemetrexed/pembrolizumab	11.3 (11.0 to 11.6)	11.3 (10.7 to 11.9)	0.1 (−0.3 to 0.4)	.72
Age, y				
66-69	11.6 (11.5 to 11.8)	11.3 (10.8 to 11.8)	1 [Reference]	NA
70-79	11.5 (11.4 to 11.6)	11.3 (10.8 to 11.8)	0.0 (−0.2 to 0.2)	.88
80-89	10.7 (10.5 to 10.9)	11.0 (10.5 to 11.6)	−0.3 (−0.5 to 0)	.04
Sex				
Male	10.8 (10.7 to 10.9)	10.9 (10.4 to 11.3)	1 [Reference]	NA
Female	12.1 (11.9 to 12.2)	11.6 (11.0 to 12.1)	0.7 (0.5 to 0.9)	<.001
Race/ethnicity				
Non-Hispanic				
White	11.4 (11.3 to 11.5)	11.0 (10.6 to 11.4)	1 [Reference]	NA
Black	11.4 (11.1 to 11.8)	11.3 (10.8 to 11.8)	0.3 (−0.1 to 0.6)	.12
Hispanic	11.1 (9.9 to 12.3)	11.1 (9.9 to 12.3)	0.1 (−1.1 to 1.3)	.86
Other	11.7 (11.3 to 12.2)	11.4 (10.8 to 12.0)	0.4 (0 to 0.9)	.06
Medicaid enrollment				
No	11.5 (11.4 to 11.6)	11.2 (10.7 to 11.7)	1 [Reference]	NA
Yes	11.0 (10.9 to 11.2)	11.2 (10.7 to 11.8)	0.1 (−0.1 to 0.3)	.56
Median household income in the census tract of residence, quintile				
Lowest 20%	11.1 (10.9 to 11.3)	11.1 (10.6 to 11.6)	1 [Reference]	NA
20%-40%	11.3 (11.1 to 11.5)	11.2 (10.6 to 11.8)	0.1 (−0.2 to 0.4)	.46
40%-60%	11.3 (11.1 to 11.5)	11.1 (10.5 to 11.7)	0.0 (−0.3 to 0.3)	.95
60%-80%	11.5 (11.3 to 11.7)	11.3 (10.7 to 11.8)	0.2 (−0.1 to 0.5)	.25
Highest 20%	11.8 (11.6 to 12.0)	11.4 (10.8 to 12.0)	0.3 (0 to 0.6)	.07
Urban/rural status in patient’s county				
Large metropolitan	11.5 (11.4 to 11.7)	11.1 (10.6 to 11.6)	1 [Reference]	NA
Metropolitan	11.4 (11.2 to 11.5)	11.1 (10.6 to 11.6)	0.0 (−0.2 to 0.2)	.79
Urban	11.1 (10.8 to 11.4)	11.1 (10.5 to 11.7)	0.1 (−0.3 to 0.4)	.78
Less urban	11.0 (10.8 to 11.3)	11.1 (10.5 to 11.7)	0.0 (−0.3 to 0.4)	.82
Rural	11.5 (11.0 to 12.0)	11.6 (10.9 to 12.3)	0.5 (0 to 1)	.07
Adults with college degree in patient’s zip code of residence, quintile				
Lowest 20%	11.0 (10.8 to 11.2)	11.0 (10.5 to 11.5)	1 [Reference]	NA
20%-40%	11.3 (11.1 to 11.5)	11.1 (10.6 to 11.7)	0.1 (−0.2 to 0.4)	.38
40%-60%	11.4 (11.2 to 11.6)	11.3 (10.7 to 11.8)	0.3 (−0.1 to 0.6)	.11
60%-80%	11.5 (11.3 to 11.7)	11.3 (10.7 to 11.9)	0.3 (−0.1 to 0.6)	.11
Highest 20%	11.7 (11.5 to 11.9)	11.4 (10.8 to 12.0)	0.4 (0 to 0.7)	.03
Date of initial treatment				
2016 first quarter	11.1 (10.7 to 11.4)	11.1 (10.7 to 11.6)	1 [Reference]	NA
2016 second quarter	11.2 (11.0 to 11.5)	11.3 (10.6 to 11.9)	0.2 (−0.3 to 0.6)	.43
2016 third quarter	11.0 (10.7 to 11.3)	11.1 (10.4 to 11.7)	−0.1 (−0.5 to 0.4)	.79
2016 fourth quarter	11.2 (10.9 to 11.5)	11.4 (10.7 to 12.0)	0.3 (−0.2 to 0.7)	.25
2017 first quarter	11.5 (11.2 to 11.7)	11.5 (10.9 to 12.2)	0.4 (0 to 0.9)	.06
2017 second quarter	11.6 (11.3 to 11.8)	11.8 (11.1 to 12.4)	0.6 (0.2 to 1.1)	.006
2017 third quarter	11.4 (11.1 to 11.7)	11.7 (11.0 to 12.3)	0.6 (0.1 to 1)	.02
2017 fourth quarter	11.2 (10.9 to 11.5)	11.4 (10.8 to 12.1)	0.3 (−0.1 to 0.8)	.16
2018 first quarter	11.8 (11.5 to 12.1)	11.9 (11.3 to 12.6)	0.8 (0.3 to 1.3)	<.001
2018 second quarter	11.5 (11.2 to 11.8)	11.7 (11.0 to 12.3)	0.5 (0.1 to 1)	.02
2018 third quarter	11.5 (11.2 to 11.8)	11.8 (11.1 to 12.4)	0.6 (0.2 to 1.1)	.006
2018 fourth quarter^a^	NA	NA	NA	NA
RSI at baseline, quintile				
Lowest 20%	14.2 (14.0 to 14.4)	13.9 (13.4 to 14.4)	1 [Reference]	NA
20%-40%	12.8 (12.6 to 13.0)	12.5 (12.0 to 13.1)	−1.4 (−1.6 to −1.1)	<.001
40%-60%	11.6 (11.4 to 11.8)	11.4 (10.9 to 12.0)	−2.5 (−2.8 to −2.2)	<.001
60%-80%	10.2 (10.0 to 10.4)	10.2 (9.6 to 10.7)	−3.7 (−4 to −3.5)	<.001
Highest 20%	8.1 (7.9 to 8.3)	8.1 (7.5 to 8.6)	−5.8 (−6.1 to −5.6)	<.001

Abbreviations: NA, not applicable; RMST, restricted mean survival time in months; RSI, Risk Stratification Index.

^a^ Too few patients from the fourth quarter of 2018 were included in the cohort to provide stable estimates.

In comparisons among treatment regimens after propensity score stratification (Table 3 and eTables 1-3 in the Supplement), patients who received pembrolizumab had similar survival (adjusted RMST, 11.0 [95% CI, 10.6-11.4] months) to those who received platinum/pemetrexed (adjusted RMST, 11.1 [95% CI, 10.9-11.3] months; adjusted RMST difference, −0.2 [95% CI, −0.5 to 0.2] months; *P* = .30). Patients who received pembrolizumab had survival that was statistically worse than that for patients who received platinum/taxane, but the magnitude of this difference was small (adjusted RMST difference, −0.7 [95% CI, −1.0 to −0.4] months; *P* < .001). Patients who received platinum/pemetrexed/pembrolizumab had survival (adjusted RMST, 11.7 [95% CI, 11.2-12.2] months) that was statistically better than that for patients who received platinum/pemetrexed (adjusted RMST, 11.2 [95% CI, 11.0-11.4] months), but the magnitude of this difference was small (adjusted RMST difference, 0.5 [95% CI, 0.1-0.9] months; *P* = .02).

**Table 3.** Propensity Score–Stratified Associations Between Treatment Regimen and Overall Survival

Comparison	No. of patients	Propensity score–adjusted results, RMST (95% CI)			*P* value
		Reference group	Pembrolizumab group	Difference	
**Pembrolizumab vs platinum/pemetrexed groups**					
Full cohort	8238	11.1 (10.9 to 11.3)	11.0 (10.6 to 11.4)	−0.2 (−0.5 to 0.2)	.30
Sensitivity^a^	5261	11.1 (10.9 to 11.4)	11.2 (10.7 to 11.7)	0.1 (−0.4 to 0.5)	.70
Men only	4125	10.1 (9.8 to 10.4)	10.8 (10.2 to 11.3)	0.7 (0.2 to 1.2)	.006
Women only	4113	12.2 (12.0 to 12.5)	11.2 (10.6 to 11.8)	−1.1 (−1.5 to −0.6)	<.001
**Pembrolizumab vs platinum/taxane groups**					
Full cohort	12 945	11.7 (11.6 to 11.9)	11.0 (10.6 to 11.3)	−0.7 (−1.0 to −0.4)	<.001
Sensitivity^a^	8351	11.8 (11.6 to 11.9)	11.2 (10.8 to 11.6)	−0.6 (−1.0 to −0.2)	.001
Men only	7143	11.1 (10.9 to 11.3)	10.6 (10.2 to 11.1)	−0.4 (−0.8 to −0.01)	.046
Women only	5802	12.5 (12.3 to 12.7)	11.4 (10.9 to 11.9)	−1.1 (−1.5 to −0.7)	<.001
**Pembrolizumab/platinum/pemetrexed vs platinum/pemetrexed groups**					
Full cohort	6584	11.2 (11.0 to 11.4)	11.7 (11.2 to 12.2)	0.5 (0.1 to 0.9)	.02
Sensitivity^a^	4198	11.3 (11.0 to 11.5)	11.7 (11.1 to 12.3)	0.4 (−0.1 to 1.0)	.15
Men only	3366	10.2 (9.9 to 10.5)	10.7 (10.0 to 11.3)	0.5 (−0.2 to 1.1)	.15
Women only	3218	12.3 (12.0 to 12.6)	12.8 (12.1 to 13.4)	0.5 (−0.2 to 1.1)	.14

Abbreviation: RMST, restricted mean survival time in months.

^a^ Includes the subset of the full cohort who had prescription medication insurance (Medicare part D) and did not receive targeted therapy (defined as treatment with any of the following drugs: gefitinib, erlotinib, afatinib, dacomitinib, osimertinib, crizotinib, ceritinib, brigatinib, alectinib, lorlatinib, dabrafenib, and trametinib).

In exploratory analyses, men who received pembrolizumab had similar overall survival to men who received platinum/pemetrexed (adjusted RMST difference, 0.7 [95% CI, 0.2-1.2] months) or men who received platinum/taxane (adjusted RMST difference, −0.4 [95% CI, −0.8 to −0.01] months). Women who received pembrolizumab had slightly worse survival than women who received platinum/pemetrexed (adjusted RMST difference, −1.1 [95% CI, −1.5 to −0.6] months) and those who received platinum/taxane (adjusted RMST difference, −1.1 [95% CI, −1.5 to −0.7] months). In an analysis stratified by age, pembrolizumab was associated with similar survival to platinum/pemetrexed across age categories, slightly worse survival with increased age compared with platinum/taxane, and slightly improved survival in the youngest age category when combined with platinum/pemetrexed (eTable 4 in the Supplement).

In sensitivity analyses restricted to the 12 549 patients with Medicare part D prescription drug coverage who did not receive oral targeted therapy within 3 months of the index claim, these associations held. However, the difference in survival outcomes between the pembrolizumab/platinum/pemetrexed chemoimmunotherapy and platinum/pemetrexed chemotherapy groups was no longer statistically significant (adjusted RMST difference, 0.4 [95% CI, −0.1 to 1.0] months; *P* = .15).

## Discussion

The incorporation of immunotherapy into the first-line treatment of older Medicare patients with advanced NSCLC was rapid after initial US Food and Drug Administration approval of pembrolizumab in November 2016; by the third quarter of 2018, more than 40% of patients in this analysis received pembrolizumab-containing regimens. In this population, patients who received immunotherapy or chemoimmunotherapy were older, were more likely to be female, and had higher baseline mortality risk than those given chemotherapy alone. Survival after first-line immunotherapy was much shorter than reported in registrational clinical trials; this finding is consistent with a recently published analysis of patients diagnosed with multiple types of cancer from 2008 to 2013,^45^ though the latter analysis cohort was too remote to assess the effectiveness of immunotherapy for lung cancer. In both multivariable- and propensity score–adjusted models that controlled for baseline differences in patients’ clinical and sociodemographic characteristics, overall survival in this cohort was similar among patients who received single-agent immunotherapy, combination chemoimmunotherapy, or chemotherapy only.

Based on clinical trial results demonstrating substantial improvements in survival with first-line immunotherapy in appropriate patients, particularly those with tumors expressing high levels of PD-L1,^25^ stronger associations between treatment regimen and overall survival might have been anticipated in this analysis. Several mechanisms could underlie the observed lack of association. First, the analysis could be vulnerable to forms of residual confounding. Although the RSI risk-adjustment score was strongly associated with outcomes in this population, patients who received immunotherapy alone may have been clinically more ill than those who received chemotherapy in ways that were not completely captured by the characteristics included in propensity models. This phenomenon could also underlie the slight heterogeneity of association between immunotherapy treatment and overall survival observed according to sex, although associations between female sex and inferior immunotherapy outcomes have also been observed in clinical trials.^25,31^

The Medicare population of patients treated for advanced NSCLC may also be substantively different from clinical trial populations. All patients in this analysis were at least 66 years of age, and the median age was 73.8, which was approximately 10 years older than the median age in KEYNOTE-024^25^ and KEYNOTE-189.^27^ The benefits of immunotherapy may require more time with treatment to emerge, yet patients may more often fail to derive any benefit than patients treated with chemotherapy, leading to poorer outcomes among older, frail patients. In trials of immunotherapy vs chemotherapy for lung cancer, particularly in populations less enriched for high-level expression of PD-L1, patients who receive immunotherapy may have similar or inferior outcomes early, and superior outcomes may emerge only among those individuals who are able to endure several months of treatment.^18,46^ In addition, single-agent programmed cell death 1 protein inhibition confers lower rates of high-grade adverse events than does chemotherapy.^17,18^ If clinicians recommend immunotherapy disproportionately to Medicare patients with poor performance status or greater comorbidity—perhaps even if PD-L1 expression levels are below thresholds associated with the most substantial immunotherapy benefit^25,46,47^—it may not be surprising that large survival improvements associated with immunotherapy were not observed in this analysis. Indeed, immunotherapy may be construed as a potential first-line treatment for patients who would otherwise have been deemed too frail for treatment at all, including patients older than 80 years. This treatment could have expanded the pool of treated patients to include those with poorer prognoses, diminishing any apparent immunotherapy benefit in a comparative effectiveness analysis. Nevertheless, expansion could improve outcomes on a population basis among all diagnosed patients by including those who might otherwise have gone untreated. Understanding the extent to which immunotherapy improves population-level outcomes by expanding the pool of treated patients will require further research.

Of note, the patients in this analysis started treatment during a period when first-line single-agent pembrolizumab was approved only for patients whose tumors exhibited PD-L1 expression of at least 50%, before the approval for patients with tumors expressing PD-L1 at a level of at least 1% based on the KEYNOTE-042 trial.^46^ Better outcomes with immunotherapy in this cohort might therefore have been anticipated. Nevertheless, durable immunotherapy benefit among some subgroups might have become more apparent with additional follow-up beyond 18 months; regardless, in both KEYNOTE-024 and KEYNOTE-189, pembrolizumab was associated with substantial improvements in overall survival by that point.^25,26,27^ Because PD-L1 results cannot be determined from Medicare claims, the extent to which pembrolizumab treatment was restricted to patients with PD-L1 expression of greater than 50% in this cohort cannot be determined.

Strengths of this analysis included its basis in the large real-world population of Medicare patients with advanced lung cancer in the US, with more contemporary treatment patterns and up-to-date survival outcomes than are currently available from the Surveillance, Epidemiology, and End Results (SEER)–Medicare linkage or the National Cancer Database. Understanding the dissemination of new cancer therapies and their effectiveness relative to established treatment approaches is critically important to achieve equity, value, and optimal outcomes. This is particularly important in oncology, because new anticancer drugs are approved after trials that include hundreds of patients—yet after approval, these drugs affect tens of thousands of patients each year, including many with frailty and multiple comorbidities who are underrepresented in clinical trials. Therefore, understanding effectiveness at the population level requires suitable data. Medicare claims are an expedient source of current data that can be used to identify emerging patterns of care and to generate hypotheses about subgroups at risk for particularly small or large benefit. Descriptions of care in the Medicare population can then be used to prioritize focus in studies that use more granular data sources, such as electronic health records or even linkages between these data and molecular profiles from next-generation sequencing.

### Limitations

The limitations of this study include the absence of key clinical and pathological data elements, which are not available in Medicare claims. Although inclusion criteria were constructed to capture patients receiving treatment indicated for advanced NSCLC, details regarding stage at diagnosis, histologic characteristics, and PD-L1 expression levels were not available; however, some of these variables may be reasonably inferred for specific cohorts. For example, pemetrexed was not approved for squamous NSCLC during any of the analysis period,^48^ such that most patients in the platinum/pemetrexed cohort likely had nonsquamous NSCLC. As above, single-agent pembrolizumab was also approved in the first-line setting only for patients with tumors expressing PD-L1 of greater than 50% during this period.^25,46^ Still, some off-label use of these drugs cannot be ruled out. Population-level databases with shorter lags to data availability are needed to better understand the effect of rapid innovation on patients with cancer; the SEER-Medicare database,^49^ for example, includes details regarding stage and histologic features, but data for patients diagnosed in 2018 are not yet available. Regardless, the survival outcomes in this cohort were similar to those observed in an analysis based on electronic health records in which detailed clinical data were available.^50^ Although a difference between survival in real-world populations and clinical trial participants could be due to competing causes of mortality in some contexts, most deaths in our cohort would be attributable to lung cancer, as observed in a recent SEER-Medicare analysis of patients with more remote diagnoses.^45^ In addition, this study focused specifically on the association between first-line therapy and outcomes, but second-line immunotherapy may well have provided benefit to some patients in the cohort who received first-line chemotherapy, diminishing the association of first-line therapy with overall survival.

## Conclusions

The findings of this retrospective cohort study constitute real-world evidence concerning the use of and survival outcomes associated with checkpoint inhibitor immunotherapy in the Medicare population of patients with advanced lung cancer. The results demonstrate rapid uptake of immunotherapy and higher rates of immunotherapy use among older patients and those with increased comorbidity. These results, and outcomes among age-, sex-, and comorbidity-level–specific subgroups, equip physicians and patients with estimates that can facilitate informed treatment decisions. Survival was much shorter than observed in registrational checkpoint inhibitor clinical trials. These results may inform prognostic considerations in practice and reinforce the importance of understanding patient selection dynamics in assessing the value and clinical utility of transformative treatment strategies.

## Notes

Supplement. **eTable 1.** Propensity Score Model: Pembrolizumab vs Platinum/Pemetrexed **eTable 2.** Propensity Score Model: Pembrolizumab vs Platinum/Taxane **eTable 3.** Propensity Score Model: Platinum/Pemetrexed/Pembrolizumab vs Platinum/Pemetrexed **eTable 4.** Propensity Score-Stratified Associations Between Treatment Regimen and Overall Survival by Age Group **eFigure 1.** Targeted Therapy Exclusion Cohort Derivation (Sensitivity Analysis) **eFigure 2.** Kaplan-Meier Overall Survival Curves by Quintile of Baseline Prediction of 1-Year Mortality at Time of Initial Treatment
