# Supplementary material for: Association Between First-Line Immune Checkpoint Inhibition and Survival for Medicare-Insured Patients With Advanced Non–Small Cell Lung Cancer
Source: JAMA Netw Open. 2021 May 21;4(5):e2111113. doi: 10.1001/jamanetworkopen.2021.11113 (PMC8140374; doi:10.1001/jamanetworkopen.2021.11113)
Supplement: Supplement. — eTable 1. Propensity Score Model: Pembrolizumab vs Platinum/Pemetrexed eTable 2. Propensity Score Model: Pembrolizumab vs Platinum/Taxane eTable 3. Propensity Score Model: Platinum/Pemetrexed/Pembrolizumab vs Platinum/Pemetrexed eTable 4. Propensity Score-Stratified Associations Between Treatment Regimen and Overall Survival by Age Group eFigure 1. Targeted Therapy Exclusion Cohort Derivation (Sensitivity Analysis) eFigure 2. Kaplan-Meier Overall Survival Curves by Quintile of Baseline Prediction of 1-Year Mortality at Time of Initial Treatment [file jamanetwopen-e2111113-s001.pdf]

Supplementary Online Content

Kehl KL, Greenwald S, Chamoun NG, Manberg PJ, Schrag D. Association between first-line immune checkpoint inhibition and survival for Medicare-insured patients with advanced non–small cell lung cancer. *JAMA Netw Open*. 2021;4(5):e2111113. doi:10.1001/jamanetworkopen.2021.11113

eTable 1. Propensity Score Model: Pembrolizumab vs Platinum/Pemetrexed

eTable 2. Propensity Score Model: Pembrolizumab vs Platinum/Taxane

eTable 3. Propensity Score Model: Platinum/Pemetrexed/Pembrolizumab vs Platinum/Pemetrexed

eTable 4. Propensity Score-Stratified Associations Between Treatment Regimen and Overall Survival by Age Group

eFigure 1. Targeted Therapy Exclusion Cohort Derivation (Sensitivity Analysis)

eFigure 2. Kaplan-Meier Overall Survival Curves by Quintile of Baseline Prediction of 1-Year Mortality at Time of Initial Treatment

This supplementary material has been provided by the authors to give readers additional information about their work.

© 2021 Kehl KL et al. *JAMA Network Open*.

eTable 1. Propensity Score Model: Pembrolizumab vs Platinum/Pemetrexed

	**Odds Ratio for Treatment with pembrolizumab vs. platinum/pemetrexed**	
**Characteristics**	**Odd Ratio Mean (95% CI*)**	**p**
**Age at the index lung cancer event**		
66-69	**Reference**	
70-79	1.32 (1.16-1.50)	<0.001
80-89	2.86 (2.44-3.35)	<0.001
**Sex**		
Male	**Reference**	
Female	1.23 (1.11-1.37)	<0.001
**Race/Ethnicity**		
White non-Hispanic	**Reference**	
Black non-Hispanic	0.83 (0.67-1.04)	0.10
Hispanic	0.86 (0.44-1.69)	0.67
Other	0.99 (0.77-1.28)	0.93
**Medicaid enrollment**		
No	**Reference**	
Yes	1.15 (1.01-1.32)	0.04
**Median household income in census tract of residence (quintile)**		
Lowest 20%	**Reference**	
20-40%	0.93 (0.78-1.12)	0.46
40-60%	0.88 (0.74-1.05)	0.16
60-80%	0.94 (0.78-1.14)	0.55
Highest 20%	0.84 (0.69-1.02)	0.08
**Urban-rural status of patient's county**		
Large metro	**Reference**	
Metro	1.18 (0.95-1.47)	0.13
Urban	1.12 (0.98-1.27)	0.09
Less urban	1.39 (0.97-1.98)	0.07
Rural	1.03 (0.82-1.30)	0.78
**% of adults with college in patient's zip code of residence (quintile)**		
Lowest 20%	**Reference**	
20-40%	1.15 (0.95-1.39)	0.15
40-60%	1.17 (0.96-1.43)	0.11
60-80%	1.23 (0.99-1.51)	0.06
Highest 20%	1.37 (1.10-1.70)	0.005
**Risk stratification index (RSI) at baseline**		
Lowest 20%	**Reference**	
20-40%	1.33 (1.12,1.59)	0.002
40-60%	1.57 (1.32,1.87)	<0.001

© 2021 Kehl KL et al. *JAMA Network Open*.

60-80%	1.86 (1.57,2.21)	<0.001
Highest 20%	2.60 (2.19,3.08)	<0.001
**Date of initial systematic lung cancer treatment**		
2016_Q1	**Reference**	
2016_Q2	**	**
2016_Q3	**	**
2016_Q4	**	**
2017_Q1	37.44 (18.32-76.52)	<0.001
2017_Q2	55.83 (27.30-114.19)	<0.001
2017_Q3	53.19 (25.97-108.95)	<0.001
2017_Q4	50.48 (24.65-103.37)	<0.001
2018_Q1	64.65 (31.60-132.26)	<0.001
2018_Q2	62.60 (30.53-128.36)	<0.001
2018_Q3	79.97 (38.93-164.29)	<0.001
2018_Q4	N/A	

** Cell information suppressed consistent with Centers for Medicare and Medicaid Services (CMS) cell size suppression policy.
N/A: Too few subjects from Q4 2018 were included in the cohort to provide stable estimates.

© 2021 Kehl KL et al. *JAMA Network Open*.

eTable 2. Propensity Score Model: Pembrolizumab vs Platinum/Taxane

	**Odds Ratio for Treatment with pembrolizumab vs. platinum/taxane**	
**Characteristics**	**Odd Ratio Mean (95% CI*)**	**p**
**Age at the index lung cancer event**		
66-69	**Reference**	
70-79	1.01 (0.90-1.14)	0.81
80-89	1.60 (1.40-1.83)	<0.001
**Sex**		
Male	**Reference**	
Female	1.66 (1.52-1.82)	<0.001
**Race/Ethnicity**		
White non-Hispanic	**Reference**	
Black non-Hispanic	0.85 (0.71-1.03)	0.09
Hispanic	1.43 (0.78-2.61)	0.25
Other	1.38 (1.09-1.73)	0.006
**Medicaid enrollment**		
No	**Reference**	
Yes	0.74 (0.66-0.83)	<0.001
**Median household income in census tract of residence (quintile)**		
Lowest 20%	**Reference**	
20-40%	0.97 (0.84-1.13)	0.71
40-60%	1.06 (0.91-1.23)	0.47
60-80%	1.13 (0.97-1.33)	0.11
Highest 20%	1.32 (1.11-1.56)	0.001
**Urban-rural status of patient's county**		
Large metro	**Reference**	
Metro	0.95 (0.79-1.14)	0.57
Urban	0.97 (0.86-1.08)	0.55
Less urban	1.03 (0.77-1.38)	0.83
Rural	0.87 (0.72-1.06)	0.16
**% of adults with college in patient's zip code of residence (quintile)**		
Lowest 20%	**Reference**	
20-40%	1.18 (1.01-1.37)	0.04
40-60%	1.27 (1.08-1.50)	0.004
60-80%	1.34 (1.12-1.60)	0.001
Highest 20%	1.73 (1.44-2.09)	<0.001
**Risk stratification index (RSI) at baseline**		
Lowest 20%	**Reference**	
20-40%	1.33 (1.13-1.55)	<0.001

© 2021 Kehl KL et al. *JAMA Network Open*.

40-60%	2.01 (1.72-2.34)	<0.001
60-80%	2.60 (2.24-3.03)	<0.001
Highest 20%	4.19 (3.60-4.87)	<0.001
**Date of initial systematic lung cancer treatment**		
2016_Q1	**Reference**	
2016_Q2	**	**
2016_Q3	**	**
2016_Q4	**	**
2017_Q1	34.32 (16.86-69.87)	<0.001
2017_Q2	39.53 (19.43-80.41)	<0.001
2017_Q3	35.55 (17.46-72.36)	<0.001
2017_Q4	40.41 (19.84-82.31)	<0.001
2018_Q1	49.98 (24.57-101.70)	<0.001
2018_Q2	39.04 (19.18-79.49)	<0.001
2018_Q3	43.01 (21.13-87.57)	<0.001
2018_Q4	N/A	

** Cell information suppressed, consistent with Centers for Medicare and Medicaid Services (CMS) cell size suppression policy.
N/A: Too few subjects from Q4 2018 were included in the cohort to provide stable estimates.

© 2021 Kehl KL et al. *JAMA Network Open*.

eTable 3. Propensity Score Model: Platinum/Pemetrexed/Pembrolizumab vs Platinum/Pemetrexed

	**Odds Ratio for Treatment with pembrolizumab/platinum/pemetrexed vs. platinum/pemetrexed**	
**Characteristics**	**Odd Ratio Mean (95% CI*)**	**p**
**Age at the index lung cancer event**		
66-69	**Reference**	
70-79	1.06 (0.90-1.23)	0.50
80-89	0.85 (0.68-1.07)	0.16
**Sex**		
Male	**Reference**	
Female	1.00 (0.87-1.14)	0.95
**Race/ethnicity**		
White non-Hispanic	**Reference**	
Black non-Hispanic	0.78 (0.59-1.04)	0.10
Hispanic	0.68 (0.25-1.83)	0.45
Other	0.82 (0.59-1.15)	0.26
**Medicaid enrollment**		
No	**Reference**	
Yes	0.92 (0.77-1.11)	0.40
**Median household income in census tract of residence (quintile)**		
Lowest 20%	**Reference**	
20-40%	1.02 (0.81-1.29)	0.86
40-60%	0.95 (0.75-1.20)	0.64
60-80%	0.99 (0.77-1.27)	0.95
Highest 20%	0.84 (0.64-1.09)	0.18
**Urban-rural status of patient's county**		
Large Metro	**Reference**	
Metro	1.15 (0.87-1.51)	0.34
Urban	0.93 (0.78-1.11)	0.41
Less Urban	1.20 (0.78-1.85)	0.41
Rural	0.98 (0.73-1.32)	0.91
**% of adults with college in patient’s zip code of residence (quintile)**		
Lowest 20%	**Reference**	
20-40%	1.04 (0.82-1.33)	0.74
40-60%	0.98 (0.76-1.27)	0.90
60-80%	1.12 (0.85-1.47)	0.41
Highest 20%	1.17 (0.88-1.55)	0.29
**Risk stratification index (RSI) at baseline**		
Lowest 20%	**Reference**	
20-40%	1.27 (1.02-1.58)	0.03

© 2021 Kehl KL et al. *JAMA Network Open*.

40-60%	1.44 (1.15-1.78)	0.001
60-80%	1.55 (1.24-1.93)	<0.001
Highest 20%	1.75 (1.40-2.19)	<0.001
**Date of initial systematic lung cancer treatment**		
2016	**Reference**	
2017	656.55 (92.19-4675.71)	<0.001
2018	N/A	

N/A: Too few subjects from Q4 2018 were included in the cohort to provide stable estimates.

© 2021 Kehl KL et al. *JAMA Network Open*.

eTable 4. Propensity Score-Stratified Associations Between Treatment Regimen and Overall Survival by Age Group

			**Propensity score-adjusted results**			
**Comparison**	**Age Cohort**	**N**	**RMST* (95% CI): Reference group**	**RMST* (95% CI): Pembrolizumab group**	**RMST* difference (95% CI)**	**p**
**Pembrolizumab vs platinum/pemetrexed**	**66-69**	2,133	10.8 (10.4-11.2)	10.7 (9.9-11.5)	-0.1 (-0.8, 0.6)	0.80
	**70-79**	4,492	11.1 (10.8-11.3)	10.9 (10.3-11.4)	-0.2 (-0.7, 0.2)	0.33
	**80-89**	1,613	10.7 (10.0-11.4)	10.2 (9.2-11.2)	-0.5 (-1.3, 0.3)	0.21
**Pembrolizumab vs platinum/taxane**	**66-69**	3,025	11.5 (11.2-11.8)	11.2 (10.5-11.9)	-0.3 (-1.0, 0.3)	0.30
	**70-79**	7,251	11.7 (11.5-11.9)	10.8 (10.4-11.3)	-0.9 (-1.3, -0.5)	<0.001
	**80-89**	2,669	11.2 (10.9-11.6)	10.5 (9.7-11.2)	-0.8 (-1.4, -0.2)	0.009
**Pembrolizumab/platinum/pemetrexed vs platinum/pemetrexed**	**66-69**	1,938	11.4 (11.0-11.8)	12.3 (11.4-13.1)	1.0 (0.1, 1.8)	0.02
	**70-79**	3,761	11.4 (11.1-11.7)	11.7 (11.1-12.3)	0.3 (-0.2, 0.9)	0.24
	**80-89**	885	10.5 (9.9-11.0)	10.5 (9.2-11.9)	0.1 (-1.1, 1.3)	0.90

© 2021 Kehl KL et al. *JAMA Network Open*.

eFigure 1. Targeted Therapy Exclusion Cohort Derivation (Sensitivity Analysis)

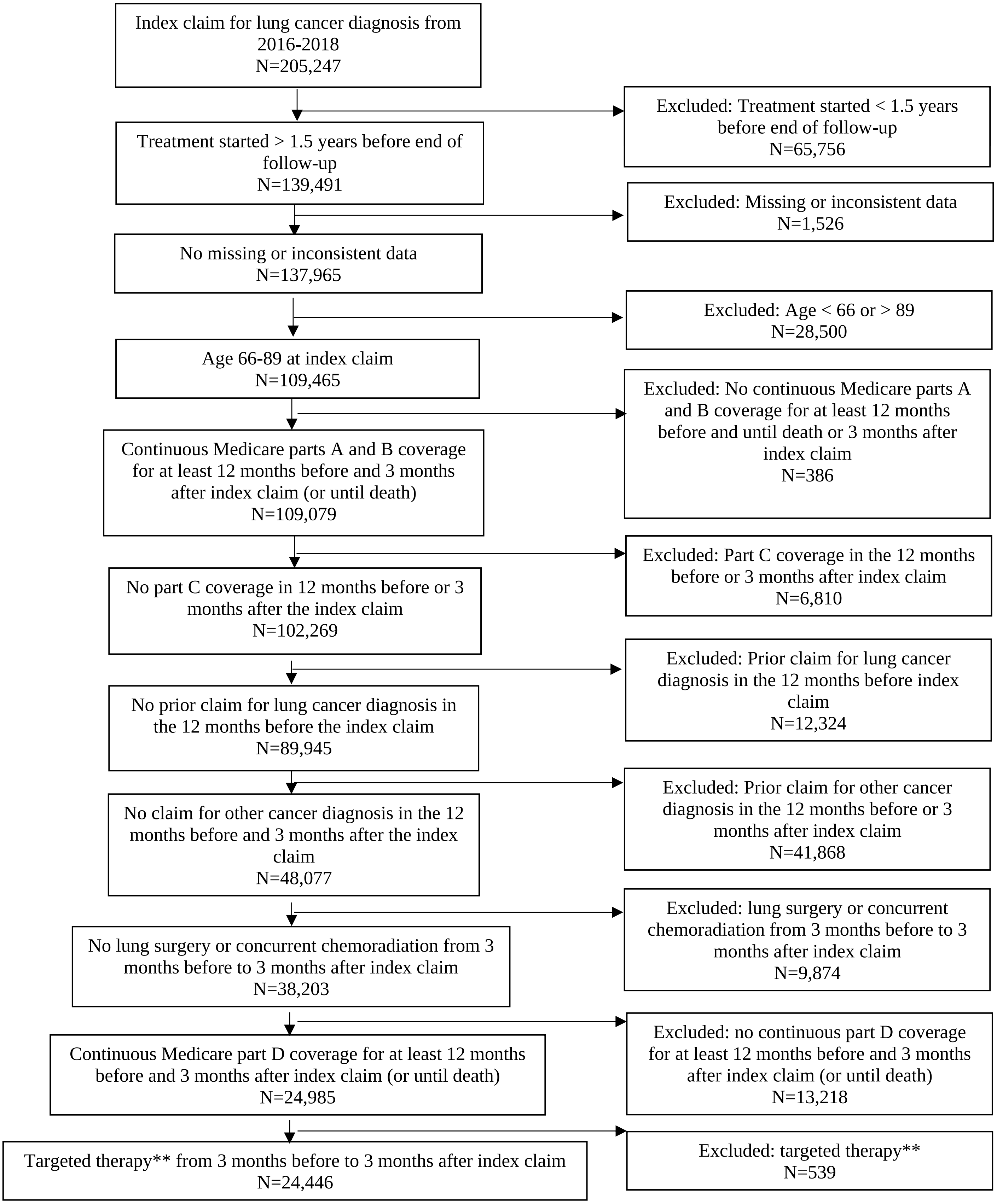

© 2021 Kehl KL et al. *JAMA Network Open*.

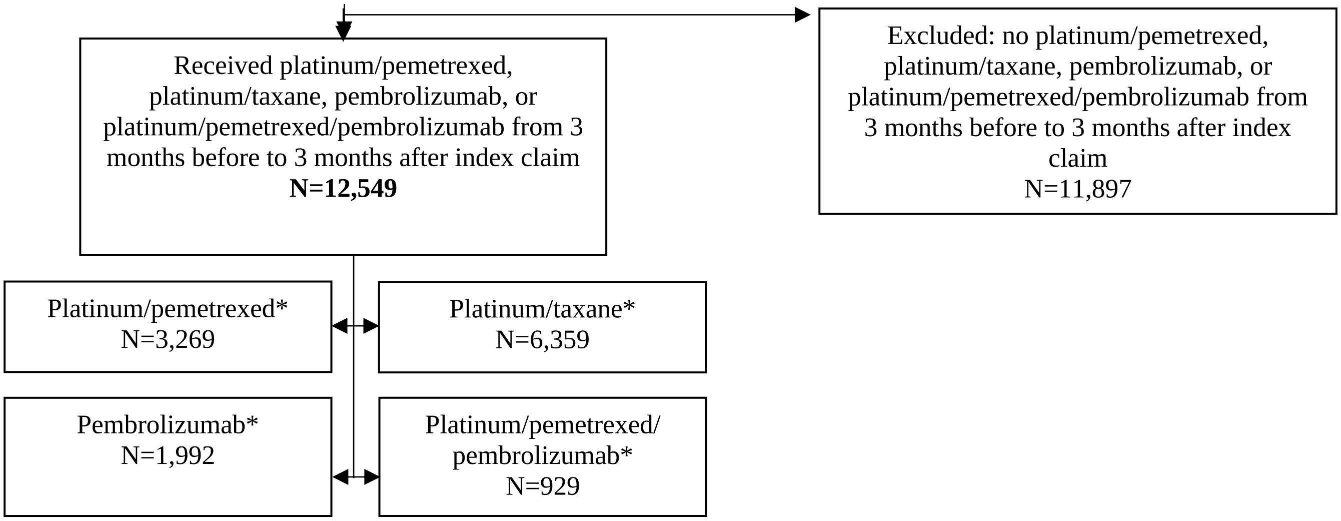

Legend to Figure S1:

* A treatment regimen was defined as any combination of chemotherapy drugs, the first dose of each of which was administered within 21 days of the first dose of the first drug given in the regimen. Platinum was defined as cisplatin or carboplatin; taxane was defined as paclitaxel, nab-paclitaxel, or docetaxel. Co-administration of bevacizumab was not considered to define a distinct regimen.

** Targeted therapy was defined as any of the following drugs: Gefitinib, erlotinib, afatinib, dacomitinib, osimertinib, crizotinib, ceritinib, brigatinib, alectinib, lorlatinib, dabrafenib, trametinib.

© 2021 Kehl KL et al. *JAMA Network Open*.

eFigure 2. Kaplan-Meier Overall Survival Curves by Quintile of Baseline Prediction of 1-Year Mortality at Time of Initial Treatment

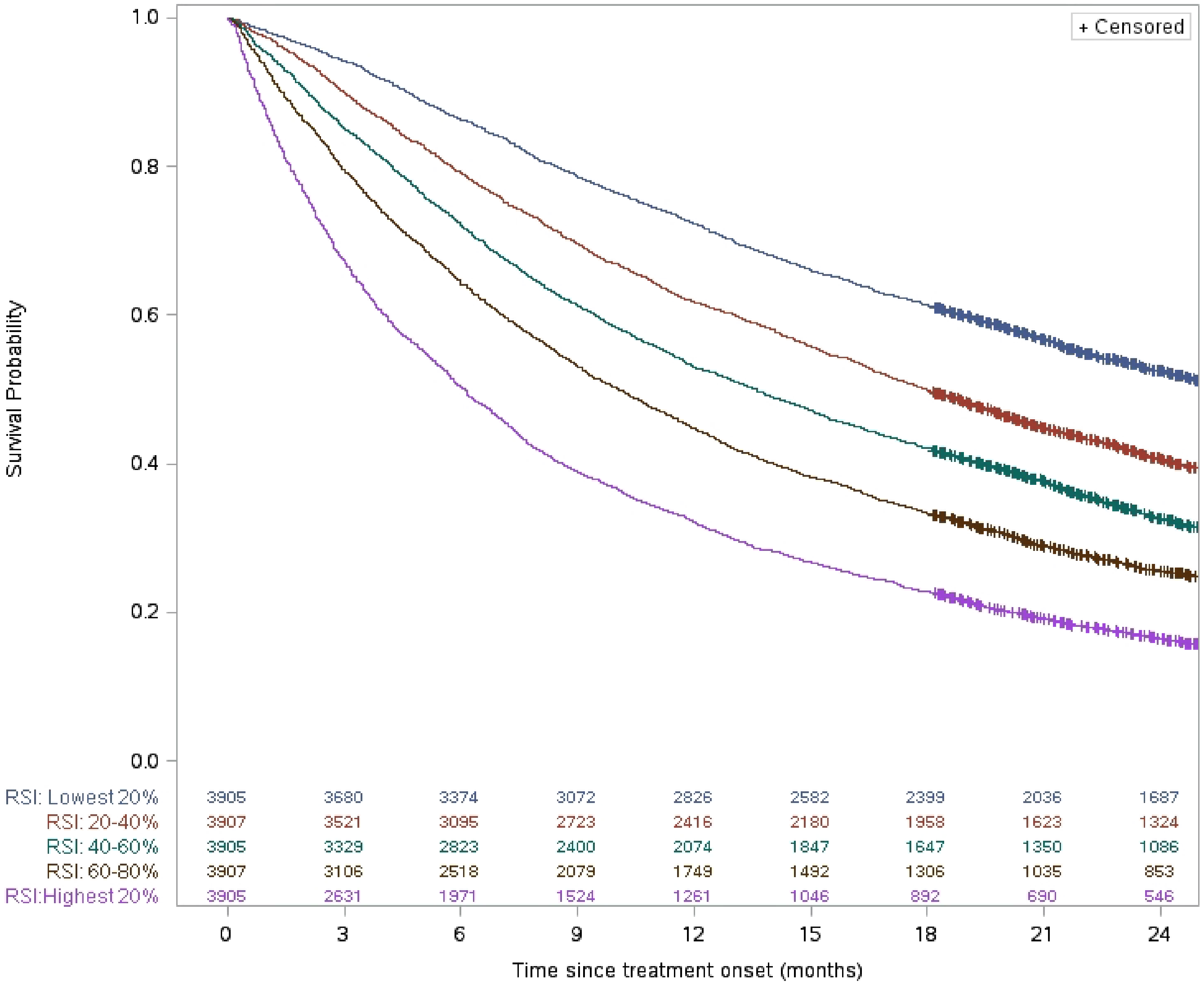

	0	3	6	9	12	15	18	21	24
RSI: Lowest 20%	3905	3680	3374	3072	2826	2582	2399	2036	1687
RSI: 20-40%	3907	3521	3095	2723	2416	2180	1958	1623	1324
RSI: 40-60%	3905	3329	2823	2400	2074	1847	1647	1350	1086
RSI: 60-80%	3907	3106	2518	2079	1749	1492	1306	1035	853
RSI:Highest 20%	3905	2631	1971	1524	1261	1046	892	690	546

	Subjects	Event	Censored	Median Survival	95% CL	
RSI: Lowest 20%	3905	2319	1586	25.8	24.7	26.7
RSI: 20-40%	3907	2721	1186	18.0	17.1	18.9
RSI: 40-60%	3905	3004	901	13.6	12.9	14.4
RSI: 60-80%	3907	3226	681	10.1	9.4	10.6
RSI:Highest 20%	3905	3473	432	6.1	5.7	6.4

Legend to Figure S2: RSI, Risk stratification index

© 2021 Kehl KL et al. *JAMA Network Open*.
